# Determination of Main Alkylamides Responsible for Numbing Sensation in Green Sichuan Pepper Through Quantitative Analysis of Multi-Components by a Single Marker with Nonivamide as an Alternative Reference Standard

**DOI:** 10.3390/foods15071143

**Published:** 2026-03-26

**Authors:** Huan Tian, Xin Li, Wei Gong, Qingxiong Yang

**Affiliations:** 1 Key Laboratory for Information System of Mountainous Areas and Protection of Ecological Environment, Guizhou Normal University, Guiyang 550025, China; tianh@gznu.edu.cn; 2 School of Chemistry and Materials Science, Guizhou Normal University, Guiyang 550025, China; 201407056@gznu.edu.cn; 3 School of Karst Science, Guizhou Normal University, Guiyang 550025, China

**Keywords:** Sichuan pepper, alkylamides, alternative reference standard method, quantitative analysis of multi-components by single marker, hydroxy-α-sanshool, nonivamide

## Abstract

The unsaturated long-chain fatty acid amide derivatives in Sichuan pepper are the components responsible for its characteristic numbing sensation. Due to the susceptibility to oxidation of these compounds, it is difficult to maintain a consistent numbing taste in pepper products, and a significant challenge is faced in the quantitative analysis of these components because the reference standards required for such analysis are very expensive, owing to the difficulty in their isolation and purification. More critically, the accuracy of determination results is always influenced by the oxidation reaction during their storage in the air. In this study, High-Performance Liquid Chromatography (HPLC) coupled with the Quantitative Analysis of Multi-components by a Single Marker (QAMS) method was employed to quantitatively analyze four main alkylamides in 28 batches of Sichuan pepper samples. The analysis revealed differences in the compositional profiles of Green Sichuan Pepper from various sources. The results obtained from both the external standard method (ESM) and the QAMS method were consistent, with standardized mean differences (SMDs) below 5.0%. Furthermore, within the QAMS method, two different internal standards, hydroxyl-α-sanshool and Nonivamide, were used. The SMD between the results obtained using these two different internal standards, respectively, was also within 5%. In conclusion, the stable and cost-effective Nonivamide can serve as a viable alternative reference standard for the HPLC-based determination of the major numbing components in Sichuan pepper.

## 1. Introduction

The genus *Zanthoxylum* in the Rutaceae family comprises over 250 species, and 45 species and 14 varieties are distributed in China [1], mainly in regions such as Shaanxi, Gansu, Sichuan, Guizhou, and Yunnan [2]. Many plants within this genus are used as traditional medicine [3], with many pharmacological activities, including local anesthesia, anti-inflammatory, analgesic, antitumor, hypoglycemic, hypolipidemic, antioxidant, and anti-infective properties [4,5,6].

The pericarps of various *Zanthoxylum* plants are widely used as condiments in Chinese cuisine. Based on traditional usage and differences in plant sources, they are categorized into two main types: Green Sichuan Pepper and Red Sichuan Pepper [7]. Red Sichuan Pepper is derived from *Zanthoxylum bungeanum*, with representative cultivars including Dahongpao, Hanyuan Huajiao, and Yuexi Gongjiao. Its fruits turn dark red or brownish-red after natural sun-drying. Green Sichuan Pepper primarily comes from *Z. armatum* and *Z. schinifolium*, featuring well-known cultivars such as Jiuyeqing from Chongqing, Tengjiao from Sichuan, and the variety *Z. planispinum var. dintanensis* from Guizhou. The fruits of green pepper remain green before and after maturity, retaining a grayish-green color after drying [8].

These different *Zanthoxylum* species are used to produce Sichuan pepper oil, ground pepper, compound seasonings, instant noodle seasoning packets, and braised products. They hold a significant position in China’s catering industry and household consumption, serving as key elements in Sichuan cuisine, Hunan cuisine, hot pot, and ma-la-tang. As the “top-tier” flavoring spice, they underpin a Mala (numbing-spicy) food industry worth hundreds of billions. The annual production and sales of Sichuan pepper in China are approximately at the level of 500,000 metric tons, showing the Sichuan pepper’s significant status in China’s seasoning industry.

**Figure 1:**
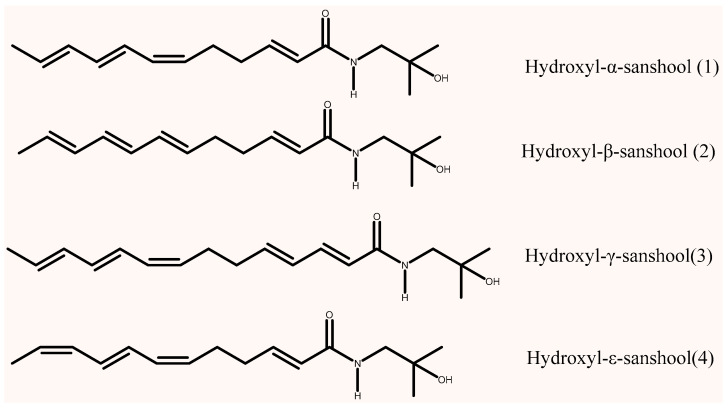
Molecular structures of compounds **1**–**4**.

“Aroma” and “numbing sensation” are the two most important quality indicators of Sichuan pepper. Red and Green Sichuan Peppers also have different culinary applications due to their distinct styles of numbing and aromatic profiles [9]. The numbing taste of Sichuan pepper results from the combined action of alkylamides, represented by hydroxyl-α-sanshool (HαSS) (Figure 1). For instance, in Green Sichuan Pepper, hydroxyl-α-sanshool is the dominant alkylamide component [10,11]. However, several other components, such as hydroxyl-β-sanshool (HβSS), hydroxyl-γ-sanshool (HγSS), and hydroxyl-ε-sanshool(HεSS), contribute differently to the numbing sensation, collectively influencing its intensity and duration [12]. These alkylamides contain multiple unsaturated bonds in their structures, making them prone to oxidation or conformational changes in air, which can lead to a reduction in or loss of the numbing taste. Therefore, the content of numbing components in Sichuan pepper and its products may fluctuate during production, storage, and circulation, leading to inconsistent product quality [13]. Thus, the quantitative determination of numbing components is the key to quality control of Sichuan pepper products.

HPLC is currently the most accurate and authoritative method for determining the numbing components in Sichuan pepper. In HPLC analysis, the target analyte is typically used as a standard reference substance [14,15,16]. Within its linear range, the content in the sample is calculated based on the relationship between signal intensity and concentration. Preparing reference standards for the alkylamides in Sichuan pepper is extremely difficult due to their structural similarity, susceptibility to oxidation, and low content, making them very expensive. Furthermore, the substances are unstable and difficult to store stably [17], leading to the formation of unknown impurities that directly compromise their purity. It is challenging to obtain stable reference standards for all numbing components. Therefore, in existing Chinese industry standards, it is recommended to use the most abundant component, HαSS, as the single standard reference substance. During calculation, the sum of peak areas of several major sanshools (HαSS, HβSS, HγSS, and HεSS) is taken as the total peak area of sanshool amides in the test sample, and the total content is calculated using the external standard method (ESM). Although this method reduces the number of standard substances required, significant errors arise from directly summing the peak areas due to the considerable differences in molar extinction coefficients among the various sanshool derivatives of different structures. Furthermore, as the contributions of different components to the numbing sensation are not identical, this method fails to accurately reflect the true numbing intensity of the pepper.

Chen et al. [18] employed the Quantitative Analysis of Multi-components by a Single Marker (QAMS) method, using HαSS as the internal standard. They determined the relative correction factors (RCFs) of other major numbing alkylamide components relative to HαSS, enabling the conversion and calculation of their respective contents. This method achieves simultaneous quantification of multiple components using a single alkylamide standard reference substance, reducing the cost of standards compared to the external standard method. However, the HαSS used as the standard still faces problems such as easy oxidation, difficult storage, and high cost [19]. Selecting readily available, stable, and inexpensive substances as alternative reference standards for expensive or unstable target analytes is a reasonable strategy to address the corresponding difficulties in the quantitative determination of Sichuan pepper’s numbing components.

In this study, Nonivamide, a stable, inexpensive, and readily available compound, was used as an alternative reference standard. A QAMS method was established for the quantitative analysis of four major numbing alkylamides in Green Sichuan Pepper. The results were simultaneously compared with those from the QAMS method using HαSS as the internal standard and the traditional ESM for the same alkylamides. Using SMD as an indicator, the deviations in the determination results of 28 batches of Sichuan pepper samples from different sources by the three methods were examined, revealing consistency among them. As the robustness and ruggedness of the QAMS method primarily depend on environmental and operational variables (such as chromatographic column, analyst, and HPLC instrument), we assessed the fluctuation and stability of the relative correction factors and investigated the effects of chromatographic column, HPLC instrument, detection wavelength, flow rate, column temperature, injection volume, and concentration of the reference standard.

## 2. Materials and Methods

### 2.1. Chemicals and Materials

HαSS, HβSS, HεSS, HγSS, and Nonivamide (≥98.00%) were purchased from Chengdu Mai De sheng Technology Co., Ltd. (Chengdu, China). Pure water was purchased from Wahaha Group Co., Ltd. (Hangzhou, China). Acetonitrile was purchased from Sigma-Aldrich (Saint Louis, MO, USA), and anhydrous ethanol was purchased from Fuyu Fine Chemical Co., Ltd. (Tianjin, China).

We collected 28 batches of Sichuan pepper samples originating from distinct geographical regions within China, and the selected plantation locations correspond to the main cultivation areas of Green Sichuan Pepper in China. The origin and producing areas of all samples are detailed in the Supplementary Table S1. The samples include 25 specimens of Green Sichuan Pepper and 3 specimens of Red Sichuan Pepper, all of which were identified uniformly by Prof. Qingxiong Yang of Guizhou Normal University. Voucher specimens have been deposited in the School of Karst Science, Guizhou Normal University. Sample No. 1 was selected for method optimization and validation.

### 2.2. Sample Preparation

Twenty-eight dry batches of Sichuan pepper samples were ground into powder, then sieved through a 40-mesh screen. An accurately weighed powdered sample (0.025 g) was transferred to a 50 mL centrifuge tube, and an appropriate amount of anhydrous ethanol was added. After being shaken thoroughly, it was extracted with the assistance of ultrasonics for 6 min in a solid-to-liquid ratio of 1:20, and a frequency of 20 kHz at 30 °C. Following extraction, the mixture was centrifuged at 2500 r min^−1^ for 10 min, and the supernatant was collected. The solid residue was subjected to a second extraction under identical conditions, and the two supernatants were combined, diluted to a final volume of 25 mL, and stored at 4 °C. Three replicates were prepared for each sample. The test solutions were obtained after being filtered through a 0.22 μm organic-based microporous membrane.

### 2.3. Preparation of Standard Solution

Five standards (HαSS, HβSS, HγSS, HεSS, and Nonivamide) were accurately weighed, placed into 10 mL volumetric flasks, and dissolved in absolute ethanol before being made up to volume. This yielded individual stock solutions with mass concentrations of 0.04, 0.10, 0.10, 0.10, and 1.01 mg mL^−1^, respectively. Appropriate volumes of each individual stock solution were accurately measured and diluted with absolute ethanol to obtain mixed standard solutions with total concentrations of 2.93, 11.73, 23.46, 29.32, and 146.60 μg mL^−1^. Prior to analysis, all solutions were preserved at −20 °C.

### 2.4. Chromatographic Conditions

Chromatographic analysis was performed using an LC-20A high-performance liquid chromatography–mass spectrometry system (Shimadzu Corporation, Kyoto, Japan) equipped with a PDA detector. A Phenomenon SuperLu C18 column (250 mm × 4.6 mm, 5 μm) (Guangzhou Phenomenon Scientific Instruments Co., Ltd., Guangzhou, China) was employed. The mobile phase consisted of water (Elution Solution A) and acetonitrile (Elution Solution B). The chromatographic column was kept at 35 °C, while the detection wavelength was adjusted to 285 nm and the flow rate was established as 1 mL min^−1^. The injection volume for both samples and standard solutions was 10 μL. Gradient elution was carried out according to the following schedule: 0–30 min, 40% B; 30–50 min, 70% B.

### 2.5. Method Validation

To evaluate the method, several key parameters were assessed: regression equation, correlation coefficient, linearity range, limit of detection (LOD), limit of quantitation (LOQ), precision, recovery, and stability.

### 2.6. Strategies for the Establishment of QAMS

Quantitative Analysis of Multi-components by a Single Marker (QAMS) method is a quantitative analytical technique that uses a single reference standard (internal standard) to simultaneously determine the concentrations of multiple components in complex samples [20]. Initially, the durability of the established method was validated, and suitable RCF values were acquired. Nonivamide and HαSS were employed as the internal reference (IR) substance for the calculation of RCFs under various experimental conditions. Afterwards, the optimal RCF was selected on the basis of the minimum relative standard deviation (RSD). Furthermore, the feasibility and reliability of the QAMS approach were further investigated using Nonivamide and HαSS. The contents of four major alkylamides in 28 batches of Sichuan pepper samples were simultaneously determined by QAMS and ESM. The QAMS method was considered acceptable when the SMD of three parallel determinations was less than 5%.

#### 2.6.1. RCF Determination and Durability Assessment

The relative correction factor (RCF) of the analyte was determined using Nonivamide and hydroxy-α-sanshool as internal standards. A mixture of reference solutions was used to evaluate two different HPLC instruments (Shimadzu LC-20A and Agilent 1260, Agilent Technologies, Santa Clara, CA, USA), chromatographic columns (Phenomenex Super Lu-C18 (250 mm × 4.6 mm, 5 μm) and AQ-C18 (150 mm × 2.1 mm, 5 μm) Shengyihongde Technology Co., Ltd., Beijing, China), flow rates (0.7, 0.9, 1.0, 1.2 mL min^−1^), column temperatures (25, 30, 35, 40 °C), injection volumes (5, 10, 15, 20 μL), and reference substance concentrations (9.67, 19.34, 24.18, 0.27, 0.54, 0.68 μg mL^−1^). The RCF value was determined by minimizing the RSD for each compound under these conditions. The final f value was calculated as the mean of the f values for all main alkylamides under the optimized conditions.

The relative correction factor (RCF) of the analyte relative to the internal standard can be calculated using Equation (1), and the concentrations of other components can then be determined using Equation (2).

(1) $$f_{is}=\frac{f_{i}}{f_{s}}=\frac{\frac{c_{i}}{A_{i}}}{\frac{c_{s}}{A_{S}}}=\frac{A_{S}\times C_{i}}{A_{i}\times C_{s}}$$

(2) $$C_{i}=f_{is}\frac{C_{s}\times A_{i}}{A_{s}}$$

In the formula: $f_{is}$: Relative correction factor; $A_{s}$: Peak area of the internal reference; $A_{i}$: Peak area of other components; $C_{s}$: Concentration of the internal reference; $C_{i}$: Analyte concentration (mg mL^−1^).

#### 2.6.2. QAMS Reproducibility Study

Based on the retention times of HαSS and Nonivamide, chromatographic peaks can be identified using the relative retention times (*RRTs*) of HεSS, HβSS, and HγSS relative to HαSS, as well as the relative retention times of HαSS, HβSS, HγSS, and HεSS relative to Nonivamide. The calculation Equation (3) is as follows:

(3) $$RRT=\frac{{RRT}_{x}}{{RRT}_{S}}$$

where *RRTx* and *RRTs* represent the retention times of the analyte and the internal reference, respectively.

Under identical chromatographic conditions, HPLC instruments (Agilent 1260, Shimadzu LC-20A) and chromatographic columns (Phenomenon SuperLu-C18 (250 mm × 4.6 mm, 5 μm) and Aq-C18 (150 mm × 2.1 mm, 5 μm)) were used to investigate the effects of two different chromatographic systems on the RRT of each component. Additionally, under identical chromatographic conditions, the Shimadzu LC-20A HPLC instrument and Phenomenon SuperLu-C18 column (250 mm × 4.6 mm, 5 μm) were employed to investigate the effects of varying column temperatures (25, 30, 40 °C), flow rates (0.7, 0.9, 1.2 mL min^−1^) and different injection volumes (5, 10, 15, 20 μL) on the RRT of each component.

#### 2.6.3. Feasibility Verification of the QAMS Method

An accurately weighed portion of Sichuan pepper sample (0.025 g) was processed via the sample preparation method described above and subsequently analyzed under the specified chromatographic conditions. Quantitation of the four analytes in each sample was performed using both ESM and QAMS. The results were subsequently compared, with the SMD calculated as an error index, to validate the feasibility of the QAMS method.

The QAMS method was compared with ESM using SMD values, which were calculated as

(4) $$SMD\left(\%\right)=\frac{\left|W_{ESM}-W_{QAMS}\right|}{W_{ESM}}\times 100\%$$

where *W_ESM_* and *W_QAMS_* are the mass contents of analytes obtained by ESM and QAMS, respectively.

(5) $$SMD\left(\%\right)=\frac{\left|W_{{QAMS}_{H\alpha SS}\;}{-W}_{{QAMS}_{Nonivamide}}\right|}{W_{{QAMS}_{H\alpha SS}}}\times 100\%$$

where *W_QAMS_* are the mass contents of analytes obtained by Hydroxy-α-sanshool (HαSS) and Nonivamide as internal standards, respectively.

### 2.7. Quantitation of Four Main Alkylamides in Sichuan Pepper

The mass content of pungent components i ($w_{i}$, mg g^−1^) was calculated as.

(6) $$W_{i}=\frac{C_{i}\times V\times K}{M_{m}}$$

where *V* is the extract volume (mL), $M_{m}$ is the sample mass (g) and *K* is the filtrate dilution factor.

### 2.8. Data Analysis

The results are presented as means ± standard deviations (SDs), calculated from triplicate analyses of each sample.

## 3. Results and Discussion

### 3.1. Optimization of Extraction Procedure

The pretreatment method basically followed the reported procedures [18], with optimization focused solely on single-factor conditions for ultrasonic-assisted extraction. Single-factor conditions were set as follows: ultrasonication time (3, 6, 10, 15, 20 min) and number of extractions (1, 2, and 3). By comparing the peak areas of target components, the optimal extraction conditions were determined as follows: solid-to-liquid ratio = 1:20, ultrasonication temperature = 30 °C, ultrasonication frequency = 20 kHz, and ultrasonication time = 6 min. After extraction, the mixture was centrifuged (2500 r min^−1^, 10 min), and the supernatant was collected. The residue underwent two additional extraction cycles, and the resulting extracts were combined.

### 3.2. Investigation of HPLC Method

#### 3.2.1. Optimization of Instrumental Conditions

**Figure 2:**
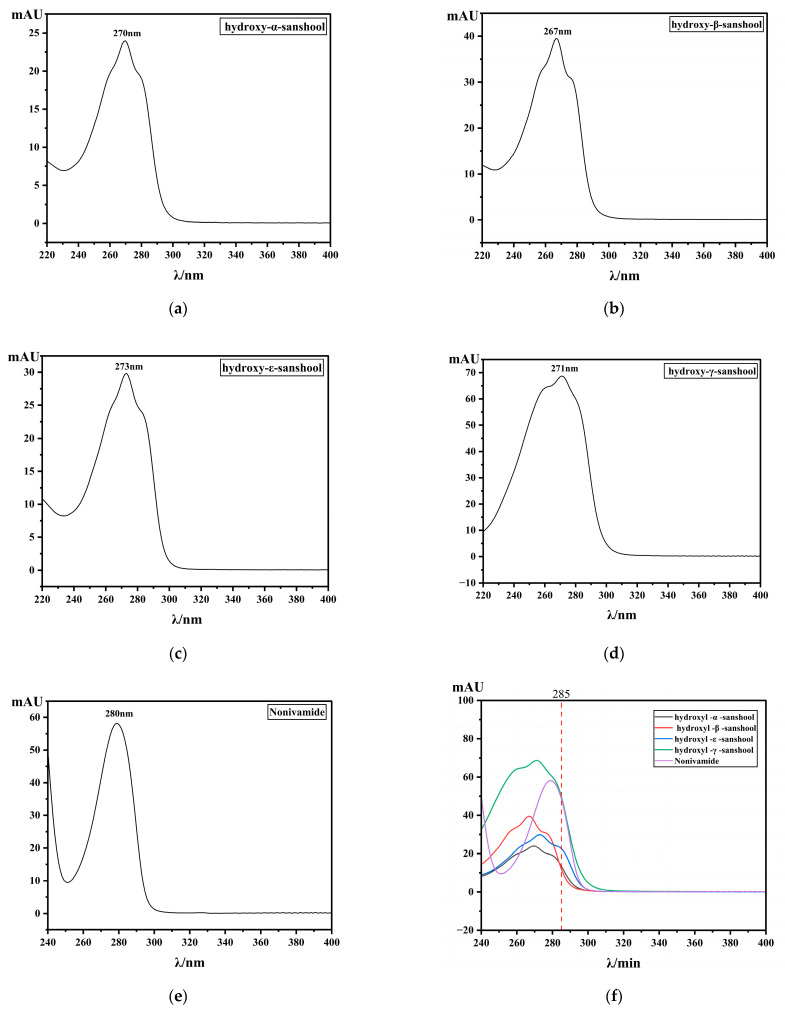
PDA scanning spectra of the five compounds (**a**–**f**).

**Figure 3:**
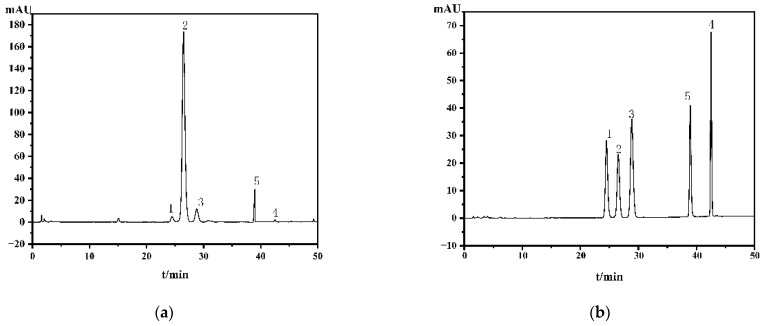
(**a**) Typical sample of Sichuan pepper; (**b**) chromatogram of the mixed standard solution: 1, hydroxyl-ε-sanshool (24 min); 2, hydroxyl-α-sanshool (26 min); 3, hydroxyl-β-sanshool (28 min); 4, hydroxyl-γ-sanshool (42 min); 5. Nonivamide (38 min) at 285 nm.

For the detection wavelength setting in HPLC-PDA analysis of the numbing compounds in Sichuan pepper, 270 nm is generally the most commonly used [21], which is closely associated with the characteristic UV absorption of these major components. As shown in Figure 2, the λ_max_ values of the five compounds all fall within the range of 268–280 nm. However, there is a certain difference between the λ_max_ (270 nm) of the four pungent components in Sichuan pepper and the λ_max_ (280 nm) of Nonivamide. When the selected wavelength leans toward the characteristic wavelength of the commonly used alkylamides, the absorption of Nonivamide decreases significantly; conversely, when the wavelength shifts toward the λ_max_ of Nonivamide, the absorption of the alkylamides is notably reduced. This is a prominent contradiction. When prioritizing the LOD and LOQ of the analytes to be determined, it can be observed that shifting the wavelength from 270 nm to 285 nm exerts little influence on the LOD and LOQ of several alkylamides (Supplementary Table S2). However, when investigating the RCFs between Nonivamide and these components, the RCF values are quite small near 270 nm, but it gradually increases as the wavelength rises. According to the guidelines for internal standard selection, to achieve higher similarity, the RCF value is usually required to be as close to 1 as possible [22]. Therefore, the detection wavelength remains at λ_max_ 270 nm in the ESM and the QAMS method with HαSS as the internal standard. However, when determining sample contents via the QAMS method with Nonivamide as the internal standard, 285 nm is selected as the detection wavelength. As illustrated in Figure 2f, all five compounds exhibit strong absorption at 285 nm, which balances both RCF and detection sensitivity. The mobile phase was optimized to achieve the optimal separation efficiency by comparing different solvents, solvent ratios, gradients (methanol-water or acetonitrile-water), and flow rates (0.3, 0.5, 0.8, 1.0, 1.5 mL min^−1^). Based on the resolution and peak areas of the target components, the optimal conditions were determined as follows: Eluent A was water, and Eluent B was acetonitrile. The column temperature was maintained at 35 °C, the flow rate was set to 1 mL min^−1^, and a gradient elution program was adopted (40% of Phase B from 0 to 30 min; 70% of Phase B from 30 to 50 min). Under the optimized conditions, the chromatograms of a typical Sichuan pepper sample and the mixed standard solution are presented in Figure 3.

#### 3.2.2. HPLC Method Validation

Plot standard calibration curves for the detector signals, with peak area as the vertical axis (Y) and reference standard mass (reference standard mass concentration × injection volume) as the horizontal axis (X). The correlation coefficients for each calibration curve were 0.9998–0.9999, indicating excellent linearity within the concentration range. Limit of quantitation (LOQ) and limit of detection (LOD) were determined at the signal-to-noise ratios of 3:1 and 10:1, respectively. All target compounds exhibited satisfactory linearity and acceptable limits of detection (LODs) and quantitation (LOQs), and the established calibration curves were validated for quantitative HPLC analysis.

A precise volume of the mixed reference solution was aspirated and injected at 10 μL. Six analyses were conducted within the same day to evaluate precision. The relative standard deviations obtained were 0.18%, 0.12%, 0.23%, 0.07%, 0.31%, indicating good instrument precision. The mixed reference solution stored at 4 °C was analyzed at 0, 1, 2, 4, 8, 10, and 24 h. The corresponding relative standard deviations were 0.41%, 0.25%, 0.38%, 0.22%, 0.28%. The test indicated no significant changes in the reference solution within 24 h. Spiked samples containing the analyte at concentrations of 80%, 100%, and 120% were analyzed. Each concentration was measured three times, yielding mean recovery rates of 97.39%, 107.00%, 98.70%, 111.60%, and 109.30%, with RSDs of 2.42%, 2.48%, 1.30%, 1.50%, and 1.10%, respectively. This demonstrates that the established method possesses reliable accuracy. The same sample was independently prepared and injected six times, yielding RSDs of 1.16%, 1.61%, 3.80%, 1.36%, 0.31%, <5%—indicating the developed method exhibits good repeatability. Results for precision, stability, repeatability, and spiked recovery are listed in Table 1.

### 3.3. Establishment of QAMS Method

#### 3.3.1. RCF Determination and Durability Assessment

**Figure 4:**
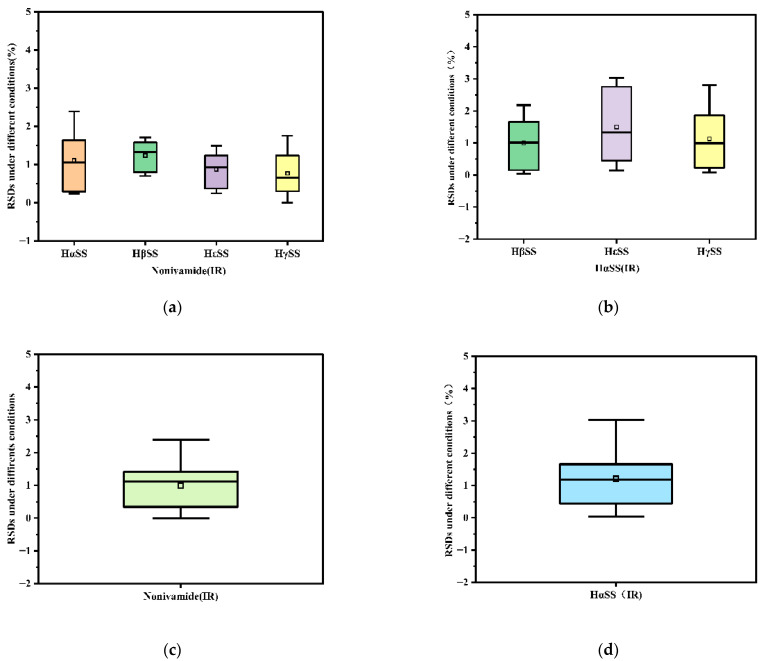
Boxplots illustrating the variations in RCF values determined by the QAMS method with two different internal references (IRs) under various conditions: RSDs of the RCF values of four target components with Nonivamide as the IR (**a**); the RSDs of the RCF values of three target components with hydroxy-α-sanshool (HαSS) as the IR (**b**); the RSDs of the RCF values of all target components with Nonivamide and hydroxy-α-sanshool as the IRs (**c**,**d**).

Using the mixed reference solution prepared with reference substance surrogates and the established analytical conditions, we calculated the relative correction factors (RCFs) between Nonivamide (as the internal reference) and the target analytes (HαSS, HβSS, HγSS, and HεSS), as well as the RCFs between hydroxy-α-sanshool (as the internal reference) and the other target analytes, followed by a systematic investigation of their robustness. Their robustness was systematically evaluated. Factors that may influence RCF were determined, with each factor selected in reference to prior research [18], including the HPLC instrument, chromatographic column, column temperature, flow rate and injection volume, and the influence of these factors was studied in detail. Under the different investigation conditions, when Nonivamide was used as the internal reference (IR), the mean relative correction factors (RCFs) with HαSS, HβSS, HεSS, and HγSS were 0.0513, 0.1321, 0.0566, and 0.0571, respectively, with the corresponding RSDs equalling 1.30%, 1.45%, 1.08%, and 0.97% (Supplementary Table S3). When hydroxy-α-sanshool served as the IR (Supplementary Table S4), the RCFs with the other analytes (HβSS, HεSS, and HγSS) were 1.6310, 1.5180, and 1.4285, respectively, with the corresponding RSDs being 0.83%, 1.57%, and 2.79%. For a more intuitive data presentation, we employed RSD boxplots (Figure 4a–d). These figures demonstrate that using the two compounds as internal references (IRs) yielded highly reproducible RCFs for all components, with RSDs below 2.76%, suggesting that the errors introduced by varying investigation conditions are minimal. Both compounds meet the IR selection criteria. However, hydroxy-α-sanshool contains multiple unsaturated bonds in its structure, which makes it prone to oxidation or configuration changes in air; this affects the purity of the standard substance, leading to issues such as difficulty in preservation and high cost. Therefore, Nonivamide—characterized by stable properties, low cost, and easy availability—was used as an alternative reference standard, as it offers the advantages of a simple structure, stability, easy accessibility, and low cost. Nonivamide was thus adopted as the alternative reference substance. Under the established chromatographic conditions, chromatographic behavior analysis revealed (Figure 3) that synthetic capsaicin (Nonivamide) and the four sanshool-type pungent components of Sichuan pepper showed comparable retention times, with narrow differences in their t_R_ values as follows: hydroxy-ε-sanshool (24 min), hydroxy-α-sanshool (26 min), hydroxy-β-sanshool (28 min), Nonivamide (38 min), and hydroxy-γ-sanshool (42 min). This result indicates a certain similarity in their chemical properties. The test results show that the RCF corresponding to Nonivamide is relatively stable, enabling the acquisition of more consistent determination results.

#### 3.3.2. Reproducibility Assessment of QAMS

Accurate peak positioning is essential for the reliable application of the QAMS method. When Nonivamide and hydroxy-α-sanshool were used as reference substances, the influence of two different brands instruments and two different model chromatographic columns on the relative retention times (RRTs) of other components was investigated to determine the peak positions (Supplementary Tables S5 and S6). The mean RRTs among the components were 1 (Nonivamide), 0.6660 (HαSS), 0.7359 (HβSS), 0.6079 (HεSS), 1.0990 (HγSS), 1 (HαSS), 1.1053 (HβSS), 0.9125 (HεSS), 1.6512 (HγSS), 0.6660 (HαSS), 0.7359 (HβSS), 0.6079 (HεSS), and 1.0990 (HγSS), respectively. These results indicate that the RRTs among components are stable across two different brands’ instruments and two different model columns and thus can be used to identify the other target compounds by referring to Nonivamide and hydroxy-α-sanshool. Different column temperatures, flow rates, and injection volumes exerted little influence on the RRTs of the pungent components in Sichuan pepper (Supplementary Tables S7 and S8).

#### 3.3.3. Similarity Evaluation of the QAMS Method and External Standard Method

The contents of HαSS, HβSS, HγSS, and HεSS in 28 batches of Sichuan pepper samples collected from different areas in China were determined using the ESM and QAMS methods (with HαSS and Nonivamide as internal standards, respectively), aiming to verify the feasibility and accuracy of the QAMS method for determining the content of numbing components. The results indicated that when Nonivamide and HαSS were used as internal standards, there were no significant differences between the contents measured by the QAMS method and the ESM (SMD < 5%) (Supplementary Tables S9 and S10). At the same time, no significant differences were observed between the results obtained by the two QAMS methods using these two different internal standards, respectively (SMD < 5%) (Supplementary Table S11). This demonstrated that the QAMS method can replace the commonly used ESM, significantly reducing the number of reference standards required in the analysis. Compared to HαSS, Nonivamide exhibits more stable chemical properties and is much more cost-effective, thereby completely overcoming the shortcomings associated with the reference standards for the numbing components in Sichuan peppers, such as high cost and chemical instability, resulting in inaccuracies in analysis due to the impurity of oxidation. Furthermore, since both numbing and spicy flavors often coexist in many products containing Sichuan peppers, using Nonivamide as a reference standard allows for the simultaneous determination of both flavor components, which is very convenient and beneficial for quality control of mala (numb-spicy) products. Although the RCF values between Nonivamide and the four main alkylamides are relatively small when Nonivamide is used as the internal standard, our determination results under different conditions showed the RCF values are rugged and robust. Moreover, its application in the analysis of multiple batches of samples was validated by both the ESM and the QAMS method using HαSS as the internal standard (which exhibits more reasonable RCF values with other components), confirming its reliability in practical applications.

### 3.4. Quantitation of Four Main Alkylamides in Sichuan Pepper

**Figure 5:**
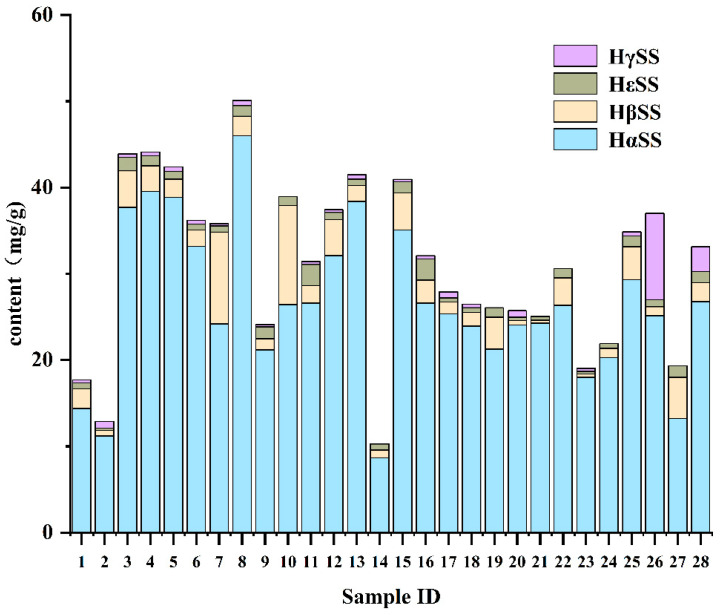
The content of four target components in 28 specimens, where the results were calculated by QAMS (Nonivamide IR).

The results of the 28 batches revealed significant differences in the content of the four numbing components among different samples: HαSS had the highest content, ranging from 11.20 ± 0.20 to 45.01 ± 0.10 mg·g^−1^, HβSS ranged from 0.34 ± 0.01 to 11.51 ± 0.08 mg·g^−1^; HεSS ranged from 0.25 ± 0.00 to 2.48 ± 0.10 mg·g^−1^; and HγSS ranged from 0.27 ± 0.00 to 10.00 ± 0.06 mg·g^−1^ (Figure 5). The proportions of the four numbing components in the total numbing components varied among different varieties, suggesting that determination of the main numbing components, respectively, is important to control the flavor of the spice. There was a significant difference in HγSS content between Red Sichuan Peppers and Green Sichuan Peppers, and there is a higher HγSS content in the former, suggesting that HγSS could serve as a marker for distinguishing between these two types. Different numbing components contribute differently to the numbing sensation, especially HγSS, which imparts a much stronger numbing effect than the others [12], suggesting why “Dahongpao,” a famous Sichuan red pepper variety, is often used as a raw material for seasonings with prominent numbing characteristics. In contrast, Green Sichuan Pepper exhibits a different numbing component profile and, due to its more unique aroma components, displays distinct flavor characteristics compared to Red Sichuan Pepper [7,23].

The total content of numbing components differed among *Zanthoxylum* samples from different producing areas, with most being more than 20 mg·g^−1^, and it was relatively high in the samples from areas of Chongqing, Yunnan, and Sichuan, the main production areas of Sichuan pepper. For instance, the total numbing component content is particularly outstanding in the samples from Jinyang, Sichuan, and from Yongshan, Yunnan, which are famous for the high-quality Sichuan green peppers. Although the highest content, with HαSS reaching up to 46 mg·g^−1^, was detected in the samples from high-altitude areas of the Daliang Mountains, Sichuan, the impact of altitude on the alkylamide content was not very pronounced. Zhenfeng County, Guizhou, is the main producing area of “Dingtan Huajiao” (*Z. planispinum* var. *dintanensis*, a variety of *Z. armatum* being regarded as a typical representative of Sichuan green peppers), and the total alkylamide contents in the samples collected in this region remains relatively stable, consistently ranging from 20 to 30 mg·g^−1^, fully demonstrating that this renowned specialty agricultural product truly lives up to its reputation.

## 4. Conclusions

Nonivamide’s application as a substitute reference standard for quantitative analysis of the numbing components in Sichuan pepper has been validated. This method exhibits excellent stability, high accuracy, high sensitivity, low cost, and simple operation. Thus, under optimized chromatographic conditions, the established QAMS method (with Nonivamide as the reference standard) is applicable for determining the contents of four target components in Sichuan pepper. This reference standard substitution approach suits quality control of numbing components in Sichuan pepper, effectively resolving the difficulties in preparing reference standards for these components (i.e., their instability and poor storage durability).

## Figures and Tables

**Table 1:** Regression equation, correlation coefficient, linear range, detection limit (LOD), quantification limit (LOQ), precision, repeatability, sample recovery rate and stability of the five compounds.

Compound	Regression Equation	Concentration Range (µg/mL)	Correlation Coefficient	LOD ng	LOQ ng	Precision (n = 6) RSD (%)	Stability (n = 0–24 h) RSD (%)	Repeatability (n = 6) RSD (%)	Recovery %	RSD (%)
HαSS	Y = 1.78 × 10^7^ X − 8686.99	0.068–85.8	0.9999	0.0680	0.2040	0.18	0.41	1.16	97.39	2.42
HβSS	Y = 1.09 × 10^7^ X − 9216.25	0.10–8.24	0.9998	0.1648	0.4940	0.12	0.25	1.61	107.00	2.48
HεSS	Y = 1.16 × 10^7^ X − 6972.88	0.10–8.00	0.9998	0.1600	0.4800	0.23	0.38	3.80	109.30	1.10
HγSS	Y = 1.20 × 10^7^ X + 1032.02	0.10–6.06	0.9999	0.1212	0.3630	0.07	0.22	1.36	111.60	1.50
Nonivamide	Y = 4.32 × 10^5^ X + 680.70	2.40–120.90	0.9999	2.4180	7.2540	0.31	0.28	0.31	98.70	1.30

## Data Availability

The original contributions presented in this study are included in the article/Supplementary Materials. Further inquiries can be directed to the corresponding authors.

## Supplementary Materials

The following supporting information can be downloaded at: https://www.mdpi.com/article/10.3390/foods15071143/s1, Table S1: Sample codes, botanical sources, and origins of 28 batches of Sichuan pepper samples, Table S2: Relative Correction Factor (RCF) values, limits of quantification (LOQ), and limits of detection (LOD) of the four target components at different wavelengths using synthetic capsaicin as the internal reference substance, Table S3: Relative correction factors (f) of the target analytes determined under different conditions using Nonivamide as the internal reference, Table S4: Relative correction factors (f) of target analytes under different conditions using hydroxy-α-sanshool as the internal reference substance, Table S5: The influence of different HPLC instruments and different model chromatographic columns on RRT when using Nonivamide as a reference, Table S6: The influence of different HPLC instruments and different model chromatographic columns on RRT when using HαSS as the reference, Table S7: Effects of Different column temperature, Flow Rate and Injection Volumes on RRT when Using Nonivamide as the Reference, Table S8: The influence of different column temperature, flow rate and injection volumes on RRT when using hydroxy-α-sanshool as the reference, Table S9: Contents of four target components (HαSS, HβSS, HεSS, HγSS) in 28 batches of Sichuan pepper samples determined using HαSS as internal standard, respectively (mg g^−1^), Table S10: Contents of four target components (hydroxy-α-sanshool, hydroxy-β-sanshool, hydroxy-ε-sanshool, hydroxy-γ-sanshool) in 28 batches of Sichuan pepper samples determined using Nonivamide as internal standard, respectively (mg g^−1^), Table S11: Comparative Analysis of the Content Results of Pungent Components in Sichuan pepper Calculated via the QAMS Method Using Hydroxy-α-sanshool (HαSS) and Nonivamide as Internal Standards (mg g^−1^).
