# Supplementary material for: Determination of Main Alkylamides Responsible for Numbing Sensation in Green Sichuan Pepper Through Quantitative Analysis of Multi-Components by a Single Marker with Nonivamide as an Alternative Reference Standard
Source: Foods. 2026 Mar 26;15(7):1143. doi: 10.3390/foods15071143 (PMC13074211; doi:10.3390/foods15071143)
Supplement: Supplementary file 1 [file foods-15-01143-s001.zip › foods-4201778-supplementary.pdf]

Supplementary Information (SI)

The supplementary material contains the following tables:

Table S1. Sample codes, botanical sources, and origins of 28 batches of Sichuan pepper samples.

Sample ID	Plant Source	Origin	Altitude (Meter)
1	*Z. armatum*	Guang an, Sichuan	700
2	*Z. armatum*	Hong ya, Sichuan	750
3	*Z. armatum*	Santai, Sichuan	500
4	*Z. armatum*	Jin yang, Sichuan	1550
5	*Z. armatum*	Ping chang, Sichuan	800
6	*Z. armatum*	Xuan han, Sichuan	950
7	*Z. armatum*	Yibin, Sichuan	450
8	*Z. armatum*	Daliang Mountains, Sichuan	2300
9	*Z. armatum*	Lu dian, Yunnan	600
10	*Z. armatum*	Yongshan, Yunnan	1200
11	*Z. armatum*	Yan yuan, Yunnan	1400
12	*Z. armatum*	Wanzhou, Chongqing	600
13	*Z. armatum*	Jiang jin, Chongqing	500
14	*Z. armatum*	De jiang, Guizhou	950
15	*Z. armatum*	Xingyi, Guizhou	1200
16	*Z. armatum*	Liu pan shui, Guizhou	900
17	*Z. planispinum var. dintanensis*	Zhen feng, Guizhou	625
18	*Z. planispinum var. dintanensis*	Zhen feng, Guizhou	625
19	*Z. planispinum var. dintanensis*	Zhen feng, Guizhou	625
20	*Z. planispinum var. dintanensis*	Zhen feng, Guizhou	725
21	*Z. planispinum var. dintanensis*	Zhen feng, Guizhou	725
22	*Z. planispinum var. dintanensis*	Zhen feng, Guizhou	725
23	*Z. planispinum var. dintanensis*	Zhen feng, Guizhou	1118
24	*Z. planispinum var. dintanensis*	Zhen feng, Guizhou	1118
25	*Z. planispinum var. dintanensis*	Zhen feng, Guizhou	1118
26	*Z . bungeanum*	Han yuan, Sichuan	1800
27	*Z . bungeanum*	Han cheng, Shaanxi	1100
28	*Z . bungeanum*	Mao wen, Sichuan	2000

Table S2. Relative Correction Factor (RCF) values, limits of quantification (LOQ), and limits of detection (LOD) of the four target components at different wavelengths using synthetic capsaicin as the internal reference substance.

Wavelength	HαSS			HβSS			HγSS			HεSS		
	RCF	LOD/ng	LOQ/ng	RCF	LOD/ng	LOQ/ng	RCF	LOD/ng	LOQ/ng	RCF	LOD/ng	LOQ/ng
254	0.0191	0.1360	0.4080	0.0324	0.3296	0.9880	0.0269	0.2424	0.7260	0.0279	0.3200	0.9600
268	0.0231	0.0680	0.2040	0.0378	0.1648	0.4940	0.0337	0.1212	0.3630	0.0357	0.1600	0.4800
270	0.0245	0.0680	0.2040	0.0401	0.1648	0.4940	0.0355	0.1212	0.3630	0.0375	0.1600	0.4800
275	0.0245	0.0680	0.2040	0.0401	0.1648	0.4940	0.0355	0.1212	0.3630	0.0375	0.1600	0.4800
280	0.0439	0.0680	0.2040	0.0805	0.1648	0.4940	0.0556	0.1212	0.3630	0.0587	0.1600	0.4800
282	0.0463	0.0680	0.2040	0.0805	0.3296	0.9880	0.0567	0.1212	0.3630	0.0591	0.1600	0.4800
285	0.05	0.068	0.2040	0.08	0.329	0.9880	0.05	0.121	0.3630	0.05	0.160	0.4800

	12	0		05	6		70	2		64	0	
290	0.06<br>14	0.136<br>0	0.4080	0.09<br>51	0.494<br>4	1.4820	0.05<br>23	0.242<br>4	0.7260	0.04<br>86	0.320<br>0	0.9600

Table S3. Relative correction factors (f) of the target analytes determined under different conditions using Nonivamide as the internal reference.

Condition	Factor	*f*-Value（Nonivamide Was Used as an Internal Standard.）			
		**HαSS**	**HβSS**	**HεSS**	**HγSS**
Column temperature /°C	25	0.0510	0.1340	0.0564	0.0571
	30	0.0510	0.1343	0.0561	0.0574
	35	0.0512	0.1305	0.0564	0.0570
	40	0.0513	0.1321	0.0563	0.0573
Flow Rates/(mL/min)	0.7	0.0523	0.1349	0.0574	0.0581
	0.9	0.0521	0.1318	0.0573	0.0574
	1.0	0.0512	0.1305	0.0564	0.0570
	1.2	0.0509	0.1344	0.0556	0.0568
Injection volume/μL	5	0.0514	0.1321	0.0561	0.0567
	10	0.0512	0.1305	0.0564	0.0570
	15	0.0515	0.1301	0.0564	0.0571
	20	0.0514	0.1303	0.0566	0.0570
Chromatographic columns	AQ-C18	0.0495	0.1281	0.0559	0.0556
	Super Lu- C18	0.0512	0.1305	0.0564	0.0570
Reference substance Concentration (μg/mL)	9.67	0.0511	0.1313	0.0570	0.0579
	19.34	0.0511	0.1325	0.0578	0.0565
	24.18	0.0518	0.1334	0.0564	0.0570
HPLC instruments	ShishizumiLC-20A	0.0512	0.1305	0.0564	0.0570
	Agilent 1260	0.0524	0.1337	0.0574	0.0570
Mean value		0.0513	0.1321	0.0566	0.0571
RSD%		1.30	1.45	1.08	0.97

Table S4. Relative correction factors (f) of target analytes under different conditions using hydroxy-α-sanshool as the internal reference substance.

Condition	Factor	*f*-value（Hydroxy-α-Sanshool Was Used as an Internal Reference Substance.）		
		**HβSS**	**HεSS**	**HγSS**
Column temperature /°C	25	1.6292	1.5035	1.4386
	30	1.6330	1.5004	1.4416
	35	1.6331	1.5276	1.4481

	40	1.6449	1.4907	1.4260
Flow Rates/(mL/min)	0.7	1.6373	1.5349	1.4524
	0.9	1.6135	1.5076	1.3817
	1.0	1.6331	1.5276	1.4481
	1.2	1.6450	1.4956	1.4494
Injection volume/μL	10	1.6331	1.5276	1.4481
	15	1.6304	1.5235	1.4500
	20	1.6353	1.5265	1.4544
Chromatographic column	AQ-C18	1.6438	1.5022	1.4766
	Super Lu- C18	1.6331	1.5276	1.4481
Reference substance concentration (μg/mL)	0.27	1.6382	1.5300	1.3376
	0.54	1.5860	1.4834	1.3545
	0.68	1.6230	1.4986	1.3639
HPLC instruments	ShishizumiLC-20A	1.6331	1.5276	1.4481
	Agilent 1260	1.6322	1.5883	1.4465
Mean value		1.6310	1.5180	1.4285
RSD%		0.83	1.57	2.79

Table S5. The influence of different HPLC instruments and different model chromatographic columns on RRT when using Nonivamide as a reference.

**HPLC Instruments**	**Chromatographic Column**	**RRT（Nonivamide Was Used as an Internal Standard.）**				
		**HαSS**	**HβSS**	**HεSS**	**HγSS**	**Nonivamide**
ShishizumiLC-20A	AQ-C18	0.6472	0.7261	0.5839	1.1155	1
	Super Lu- C18	0.6831	0.7430	0.6309	1.0921	1
Agilent 1260	AQ-C18	0.6542	0.7345	0.5900	1.0955	1
	Super Lu- C18	0.6796	0.7401	0.6267	1.0928	1
Mean value		0.6660	0.7359	0.6079	1.0990	1
RSD（%）		2.70	1.01	4.00	1.01	0

Table S6. The influence of different HPLC instruments and different model chromatographic columns on RRT when using HαSS as the reference.

**HPLC Instruments**	**Chromatographic Column**	**RRT（HαSS Was Used as an Internal Reference Substance.）**			
		**HαSS**	**HβSS**	**HεSS**	**HγSS**
ShishizumiLC-20A	AQ-C18	1	1.1219	0.9023	1.7236

	SuperLu- C18	1	1.0877	0.9236	1.5987
Agilent 1260	AQ-C18	1	1.1227	0.9019	1.6744
	SuperLu- C18	1	1.0890	0.9221	1.6080
Mean value		1	1.1053	0.9125	1.6512
RSD（%）		0	1.77	1.32	3.57

Table S7. Effects of Different column temperature, Flow Rate and Injection Volumes on RRT when Using Nonivamide as the Reference.

**Condition**	**Factor**	**RRT-Value（Nonivamide Was Used as an Internal Standard.）**			
		**HαSS**	**HβSS**	**HεSS**	**HγSS**
Column temperature /°C	25	0.6692	0.7302	0.6166	1.0748
	30	0.6772	0.7378	0.6247	1.0836
	40	0.6826	0.7413	0.6313	1.1020
	Mean value	0.6764	0.7364	0.6242	1.0868
	RSD%	0.99	0.77	1.18	1.28
Injection volume/μL	5	0.6841	0.7439	0.6318	1.0918
	10	0.6831	0.7430	0.6309	1.0921
	15	0.6837	0.7436	0.6314	1.0921
	20	0.6830	0.7429	0.6308	1.0925
	Mean value	0.6835	0.7434	0.6312	1.0921
	RSD%	0.07	0.07	0.07	0.03
Flow Rates/(mL/min)	0.7	0.7060	0.8648	0.627569	1.0940
	0.9	0.7238	0.7871	0.643857	1.0806
	1.2	0.6840	0.6779	0.591945	1.1050
	Mean value	0.7046	0.7766	0.6211	1.0932
	RSD%	2.83	3.26	4.27	1.12

Table S8. The influence of different column temperature, flow rate and injection volumes on RRT when using hydroxy-α-sanshool as the reference.

**Condition**	**Factor**	**RRT-Value（Hydroxy-α-Sanshool Was Used as an Internal Reference Substance.）**			
		**HαSS**	**HβSS**	**HεSS**	**HγSS**
Column temperature /°C	25	1	1.0912	0.9213	1.6060
	30	1	1.0895	0.9224	1.5999

	40	1	1.0860	0.9249	1.6144
Injection volume/μL	10	1	1.0877	0.9236	1.5987
	15	1	1.0877	0.9235	1.5974
	20	1	1.0877	0.9235	1.5995
Flow Rates /(mL/min)	0.7	1	1.1102	0.8888	1.5494
	0.9	1	1.0875	0.8895	1.4929
	1.2	1	1.0848	0.8654	1.6155
Mean value		1	1.0903	0.9092	1.5860
RSD%		0	0.71	2.43	2.52

Table S9. Contents of four target components (HαSS, HβSS, HεSS, HγSS) in 28 batches of Sichuan pepper samples determined using HαSS as internal standard, respectively (mg g-1).

Sample Number	Internal Standard			Pungent Compounds								
	HαSS			HβSS			HεSS			HγSS		
	ESM	QAMS	SMD%	ESM	QAMS	SMD%	ESM	QAMS	SMD%	ESM	QAMS	SMD%
1	13.97 ±0.20	13.97 ±0.20	0.00	2.27 ±0.03	2.26 ±0.03	0.44	0.69 ±0.00	0.69 ±0.00	0.00	0.37 ±0.00	0.37 ±0.00	0.00
2	10.78 ±0.20	10.78 ±0.20	0.00	0.68 ±0.01	0.68 ±0.01	0.00	0.26 ±0.00	0.26 ±0.00	0.00	0.81 ±0.00	0.81 ±0.00	0.00
3	36.23 ±0.10	36.23 ±0.10	0.00	4.30 ±0.03	4.28 ±0.03	0.47	1.59 ±0.01	1.58 ±0.01	0.63	0.42 ±0.00	0.41 ±0.00	2.38
4	38.23 ±0.06	38.23 ±0.06	0.00	3.02 ±0.03	3.00 ±0.03	0.66	1.20 ±0.00	1.19 ±0.00	0.83	0.42 ±0.00	0.43 ±0.00	2.38
5	37.76 ±0.05	37.76 ±0.05	0.00	2.19 ±0.01	2.18 ±0.03	0.46	0.92 ±0.00	0.92 ±0.00	0.00	0.54 ±0.00	0.52 ±0.00	3.70
6	32.35 ±0.08	32.35 ±0.08	0.00	1.95 ±0.03	1.93 ±0.03	1.03	0.72 ±0.02	0.72 ±0.02	0.00	0.45 ±0.00	0.43 ±0.00	4.44
7	21.00 ±0.04	21.00 ±0.04	0.00	10.66 ±0.07	10.61 ±0.07	0.47	2.49 ±0.10	2.46 ±0.10	1.20	0.27 ±0.00	0.26 ±0.00	3.70
8	31.09 ±0.10	31.09 ±0.10	0.00	2.32 ±0.01	2.31 ±0.01	0.43	0.58 ± 0.01	0.59 ±0.01	1.72	0.60±0.00	0.59 ±0.00	1.67
9	34.54 ±0.10	34.54 ±0.10	0.00	1.34 ±0.01	1.33 ±0.01	0.75	0.26 ±0.01	0.27 ±0.01	3.85	0.27 ±0.00	0.26 ±0.00	3.70
10	36.25 ±0.08	36.25 ±0.08	0.00	11.52 ±0.08	11.47 ±0.08	0.43	2.49 ±0.01	2.46 ±0.01	1.20	-	-	-
11	25.74 ±0.6	25.74 ±0.6	0.00	2.08 ±0.01	2.07 ±0.01	0.48	0.76 ±0.02	0.76 ±0.02	0.00	0.31 ±0.00	0.32 ±0.00	3.23
12	25.45 ±0.04	25.45 ±0.04	0.00	4.23 ±0.01	4.21 ±0.01	0.47	1.29 ±0.02	1.29 ±0.02	0.00	0.32 ±0.00	0.31 ±0.00	3.13
13	25.61 ±0.4	25.61 ±0.4	0.00	1.90 ±0.03	1.89 ±0.03	0.53	0.84 ±0.01	0.84 ±0.01	0.00	0.51 ±0.00	0.51 ±0.00	0.00
14	26.21 ±0.07	26.21 ±0.07	0.00	0.98 ±0.01	0.98 ±0.01	0.00	0.32 ±0.00	0.33 ±0.00	3.13	-	-	-
15	24.35 ±0.30	24.35 ±0.30	0.00	4.35 ±0.04	4.33 ±0.04	0.46	1.41 ±0.03	1.41 ±0.03	0.00	0.27 ±0.00	0.26 ±0.00	3.70
16	22.74 ±0.50	22.74 ±0.50	0.00	2.72 ±0.01	2.70 ±0.01	0.74	0.87 ±0.01	0.88 ±0.01	1.15	0.36 ±0.00	0.35 ±0.00	2.78
17	24.58 ±0.10	24.58 ±0.10	0.00	1.42 ±0.01	1.41 ±0.01	0.70	0.50 ±0.00	0.51 ±0.00	2.00	0.70 ±0.00	0.69 ±0.00	1.43
18	23.19 ±0.09	23.19 ±0.09	0.00	1.64 ±0.01	1.63 ±0.01	0.61	0.54 ±0.00	0.55 ±0.00	1.85	0.40 ±0.00	0.39 ±0.00	2.50
19	20.60 ±0.10	20.60 ±0.10	0.00	3.70 ±0.01	3.68 ±0.01	0.54	1.12 ±0.01	1.12 ±0.01	0.00	-	-	-
20	23.32 ±0.10	23.32 ±0.10	0.00	0.53 ±0.01	0.52 ±0.01	1.89	0.38 ±0.00	0.38 ±0.00	0.00	0.77 ±0.00	0.75 ±0.00	2.60
21	23.51 ±0.06	23.51 ±0.06	0.00	0.35 ±0.01	0.34 ±0.01	2.86	0.45 ±0.00	0.46 ±0.00	2.22	-	-	-
22	25.50 ±0.06	25.50 ±0.06	0.00	3.22 ±0.01	3.21 ±0.01	0.31	1.10 ±0.00	1.10 ±0.00	0.00	-	-	-
23	17.45 ±0.40	17.45 ±0.40	0.00	0.42 ±0.01	0.42 ±0.01	0.00	0.30 ±0.00	0.30 ±0.00	0.00	0.39 ±0.00	0.38 ±0.00	2.56
24	19.62 ±0.03	19.62 ±0.03	0.00	1.15 ±0.01	1.14 ±0.01	0.87	0.57 ±0.00	0.57 ±0.00	0.00	-	-	-
25	28.40±0.09	28.40±0.09	0.00	3.81 ±0.03	3.80 ±0.03	0.26	1.31 ±0.00	1.31 ±0.00	0.00	0.45 ±0.00	0.43 ±0.00	4.44
26	36.72 ±0.06	36.72 ±0.06	0.00	1.10 ±0.01	1.09 ±0.01	0.91	0.34 ±0.00	0.35 ±0.00	2.94	9.87 ±0.06	10.11 ±0.06	2.43
27	16.02 ±0.10	16.02 ±0.10	0.00	4.79 ±0.01	4.77 ±0.01	0.42	1.32 ±0.01	1.31 ±0.01	0.76	-	-	-
28	13.17 ±0.10	13.17 ±0.10	0.00	2.27 ±0.01	2.26 ±0.01	0.44	0.73 ±0.03	0.73 ±0.03	0.00	2.87 ±0.03	2.89 ±0.03	0.70

Table S10. Contents of four target components (hydroxy-α-sanshool, hydroxy-β-sanshool, hydroxy-ε-sanshool, hydroxy-γ-sanshool) in 28 batches of Sichuan pepper samples determined using Nonivamide as internal standard, respectively (mg g-1).

Sample Number	Pungent Compounds											
	HαSS			HβSS			H ε SS			H γ SS		
	ESM	QAMS	SMD%	ESM	QAMS	SMD%	ESM	QAMS	SMD%	ESM	QAMS	SMD%
1	13.97 ±0.20	14.41 ±0.20	3.15	2.27 ±0.03	2.26 ±0.03	0.44	0.69 ±0.00	0.69 ±0.00	0.00	0.37 ±0.00	0.38 ±0.00	2.70
2	10.78 ±0.20	11.20 ±0.20	3.90	0.68 ±0.01	0.66 ±0.01	2.94	0.26 ±0.00	0.25 ±0.00	3.85	0.81 ±0.00	0.80 ±0.00	1.23
3	36.23 ±0.10	37.69 ±0.10	4.03	4.30 ±0.03	4.25 ±0.03	1.16	1.59 ±0.01	1.57 ±0.01	1.26	0.42 ±0.00	0.41 ±0.00	2.50
4	38.23 ±0.06	39.56 ±0.06	3.48	3.02 ±0.03	2.96 ±0.03	1.99	1.20 ±0.00	1.17 ±0.00	2.50	0.42 ±0.00	0.42 ±0.00	2.44
5	37.76 ±0.05	38.85 ±0.05	2.89	2.19 ±0.01	2.13 ±0.01	2.74	0.92 ±0.00	0.88 ±0.00	4.35	0.54 ±0.00	0.52 ±0.00	1.96
6	32.35 ±0.08	33.19 ±0.08	2.60	1.95 ±0.03	1.88 ±0.03	3.59	0.72 ±0.02	0.73 ±0.02	1.39	0.45 ±0.00	0.44 ±0.00	2.33
7	21.00 ±0.04	23.34 ±0.04	2.64	10.66 ±0.07	10.65 ±0.07	0.09	2.49 ±0.10	2.48 ±0.10	0.40	0.27 ±0.00	0.27 ±0.00	3.85
8	31.09 ±0.10	45.01 ±0.10	2.93	2.32 ±0.01	2.26 ±0.01	2.59	0.58 ± 0.01	0.58 ±0.01	0.00	0.60±0.00	0.59 ±0.00	0.00
9	34.54 ±0.10	21.20 ±0.10	1.97	1.34 ±0.01	1.35 ±0.01	1.03	0.26 ±0.01	0.26 ±0.01	0.00	0.27 ±0.00	0.27 ±0.00	3.85
10	36.25 ±0.08	26.42 ±0.08	2.64	11.52 ±0.08	11.51 ±0.08	0.09	2.49 ±0.01	2.48 ±0.01	0.40	-	-	-
11	25.74 ±0.60	26.60±0.60	3.87	2.08 ±0.01	2.03 ±0.01	2.40	0.76 ±0.02	0.73 ±0.02	3.95	0.31 ±0.00	0.31 ±0.00	0.00
12	25.45 ±0.04	32.11 ±0.04	3.28	4.23 ±0.01	4.17 ±0.01	1.42	1.29 ±0.02	1.26 ±0.02	2.33	0.32 ±0.00	0.32 ±0.00	3.23
13	25.61 ±0.40	38.4 ±0.40	4.58	1.90 ±0.03	1.83 ±0.03	3.68	0.84 ±0.01	0.80±0.01	4.76	0.51 ±0.00	0.52 ±0.00	1.96
14	26.21 ±0.07	8.47 ±0.07	2.92	0.98 ±0.01	0.98 ±0.01	0.33	0.32 ±0.00	0.32 ±0.00	0.00	-	-	-
15	24.35 ±0.30	35.08 ±0.30	1.56	4.35 ±0.04	4.30 ±0.04	1.15	1.41 ±0.03	1.43 ±0.03	1.42	0.27 ±0.00	0.27 ±0.00	3.85
16	22.74 ±0.50	26.59 ±0.50	4.48	2.72 ±0.01	2.66 ±0.01	2.21	0.87 ±0.01	0.85 ±0.01	2.30	0.36 ±0.00	0.35 ±0.00	2.94
17	24.58 ±0.10	25.36 ±0.10	3.17	1.42 ±0.01	1.37 ±0.01	3.52	0.50 ±0.00	0.49 ±0.00	2.00	0.70 ±0.00	0.70 ±0.00	0.00
18	23.19 ±0.09	23.95 ±0.09	3.28	1.64 ±0.01	1.59 ±0.01	3.05	0.54 ±0.00	0.53 ±0.00	1.85	0.40 ±0.00	0.40 ±0.00	0.00
19	20.60 ±0.10	21.28 ±0.10	3.30	3.70 ±0.01	3.69 ±0.01	0.27	1.12 ±0.01	1.12 ±0.01	0.00	-	-	-
20	23.32 ±0.10	24.06 ±0.10	3.17	0.53 ±0.01	0.53 ±0.01	0.00	0.38 ±0.00	0.37 ±0.00	2.63	0.77 ±0.00	0.77 ±0.00	0.00
21	23.51 ±0.06	24.28 ±0.06	3.28	0.35 ±0.01	0.34 ±0.01	2.43	0.45 ±0.00	0.45 ±0.00	0.00	-	-	-
22	25.50 ±0.06	26.33 ±0.06	3.25	3.22 ±0.01	3.20 ±0.01	0.62	1.10 ±0.00	1.07 ±0.00	2.73	-	-	-
23	17.45 ±0.40	17.99 ±0.40	3.09	0.42 ±0.01	0.40 ±0.01	4.76	0.30 ±0.00	0.30 ±0.00	0.00	0.39 ±0.00	0.38 ±0.00	2.56
24	19.62 ±0.03	20.27 ±0.03	3.31	1.15 ±0.01	1.15 ±0.01	0.34	0.57 ±0.00	0.55 ±0.00	3.51	-	-	-
25	28.40±0.09	29.31 ±0.09	3.20	3.81 ±0.03	3.81 ±0.03	0.00	1.31 ±0.00	1.29 ±0.00	1.53	0.45 ±0.00	0.45 ±0.000	0.00
26	36.72 ±0.06	25.17 ±0.06	3.84	1.10 ±0.01	1.12 ±0.01	2.08	0.34 ±0.00	0.34 ±0.00	0.00	9.87 ±0.06	10 .00±0.06	1.09
27	16.02 ±0.10	13.24 ±0.10	0.53	4.79 ±0.01	4.74 ±0.01	1.04	1.32 ±0.01	1.36 ±0.01	3.03	-	-	-
28	13.17 ±0.10	26.76 ±0.10	2.10	2.27 ±0.01	2.21 ±0.01	2.64	0.73 ±0.03	0.70 ±0.03	4.11	2.87 ±0.03	2.86 ±0.03	1.04

Table S11. Comparative Analysis of the Content Results of Pungent Components in Sichuan pepper Calculated via the QAMS Method Using Hydroxy-α-sanshool (HαSS) and Nonivamide as Internal Standards (mg g-1).

	HαSS			HβSS			HεSS			HγSS		
**Internal Standards**	**HαSS**	**Nonivamide**		**HαSS**	**Nonivamide**		**HαSS**	**Nonivamide**		**HαSS**	**Nonivamide**	
**Sample Number**	**QAMS**	**QAMS**	**SMD%**	**QAMS**	**QAMS**	**SMD%**	**QAMS**	**QAMS**	**SMD%**	**QAMS**	**QAMS**	**SMD%**
1	13.97 ±0.20	14.41 ±0.20	3.15	2.26 ±0.03	2.26 ±0.03	0.00	0.69 ±0.00	0.69 ±0.00	0.00	0.37 ±0.00	0.38 ±0.00	2.70
2	10.78 ±0.20	11.20 ±0.20	3.90	0.68 ±0.01	0.66 ±0.01	2.94	0.26 ±0.00	0.25 ±0.00	3.85	0.81 ±0.00	0.80 ±0.00	1.23
3	36.23 ±0.10	37.69 ±0.10	4.03	4.28 ±0.03	4.25 ±0.03	0.70	1.58 ±0.01	1.57 ±0.01	0.63	0.41 ±0.00	0.41 ±0.00	0.00
4	38.23 ±0.06	39.56 ±0.06	3.48	3.00 ±0.03	2.96 ±0.03	1.33	1.19 ±0.00	1.17 ±0.00	1.68	0.43 ±0.00	0.42 ±0.00	2.33
5	37.76 ±0.05	38.85 ±0.05	2.89	2.18 ±0.03	2.13 ±0.01	2.29	0.92 ±0.00	0.88 ±0.00	4.35	0.52 ±0.00	0.52 ±0.00	0.00
6	32.35 ±0.08	33.19 ±0.08	2.60	1.93 ±0.03	1.88 ±0.03	2.59	0.72 ±0.02	0.73 ±0.02	1.39	0.43 ±0.00	0.44 ±0.00	2.33
7	21.00 ±0.04	23.34 ±0.04	2.64	10.61 ±0.07	10.65 ±0.07	0.38	2.46 ±0.10	2.48 ±0.10	0.81	0.26 ±0.00	0.27 ±0.00	3.85
8	31.09 ±0.10	45.01 ±0.10	2.93	2.31 ±0.01	2.26 ±0.01	2.16	0.59 ±0.01	0.58 ±0.01	1.69	0.59 ±0.00	0.59 ±0.00	0.00
9	34.54 ±0.10	21.20 ±0.10	1.97	1.33 ±0.01	1.35 ±0.01	1.50	0.27 ±0.01	0.26 ±0.01	3.70	0.26 ±0.00	0.27 ±0.00	3.85
10	36.25 ±0.08	26.42 ±0.08	2.64	11.47 ±0.08	11.51 ±0.08	0.35	2.46 ±0.01	2.48 ±0.01	0.81	-	-	-
11	25.74 ±0.60	26.60±0.60	3.87	2.07 ±0.01	2.03 ±0.01	1.93	0.76 ±0.02	0.73 ±0.02	3.95	0.32 ±0.00	0.31 ±0.00	3.13
12	25.45 ±0.04	32.11 ±0.04	3.28	4.21 ±0.01	4.17 ±0.01	0.95	1.29 ±0.02	1.26 ±0.02	2.33	0.31 ±0.00	0.32 ±0.00	3.23
13	25.61 ±0.40	38.4 ±0.40	4.58	1.89 ±0.03	1.83 ±0.03	3.17	0.84 ±0.01	0.80±0.01	4.76	0.51 ±0.00	0.52 ±0.00	1.96
14	26.21 ±0.07	8.47 ±0.07	2.92	0.98 ±0.01	0.98 ±0.01	0.00	0.33 ±0.00	0.32 ±0.00	3.03	-	-	-
15	24.35 ±0.30	35.08 ±0.30	1.56	4.33 ±0.04	4.30 ±0.04	0.69	1.41 ±0.03	1.43 ±0.03	1.42	0.26 ±0.00	0.27 ±0.00	3.85
16	22.74 ±0.50	26.59 ±0.50	4.48	2.70 ±0.01	2.66 ±0.01	1.48	0.88 ±0.01	0.85 ±0.01	3.41	0.35 ±0.00	0.35 ±0.00	0.00
17	24.58 ±0.10	25.36 ±0.10	3.17	1.41 ±0.01	1.37 ±0.01	2.84	0.51 ±0.00	0.49 ±0.00	3.92	0.69 ±0.00	0.70 ±0.00	1.45
18	23.19 ±0.09	23.95 ±0.09	3.28	1.63 ±0.01	1.59 ±0.01	2.45	0.55 ±0.00	0.53 ±0.00	3.64	0.39 ±0.00	0.40 ±0.00	2.56
19	20.60 ±0.10	21.28 ±0.10	3.30	3.68 ±0.01	3.69 ±0.01	0.27	1.12 ±0.01	1.12 ±0.01	0.00	-	-	-
20	23.32 ±0.10	24.06 ±0.10	3.17	0.52 ±0.01	0.53 ±0.01	1.92	0.38 ±0.00	0.37 ±0.00	2.63	0.75 ±0.00	0.77 ±0.00	2.67
21	23.51 ±0.06	24.28 ±0.06	3.28	0.34 ±0.01	0.34 ±0.01	0.00	0.46 ±0.00	0.45 ±0.00	2.17	-	-	-
22	25.50 ±0.06	26.33 ±0.06	3.25	3.21 ±0.01	3.20 ±0.01	0.31	1.10 ±0.00	1.07 ±0.00	2.73	-	-	-
23	17.45 ±0.40	17.99 ±0.40	3.09	0.42 ±0.01	0.40 ±0.01	4.76	0.30 ±0.00	0.30 ±0.00	0.00	0.38 ±0.00	0.38 ±0.00	0.00
24	19.62 ±0.03	20.27 ±0.03	3.31	1.14 ±0.01	1.15 ±0.01	0.88	0.57 ±0.00	0.55 ±0.00	3.51	-	-	-
25	28.40±0.09	29.31 ±0.09	3.20	3.80 ±0.03	3.81 ±0.03	0.26	1.31 ±0.00	1.29 ±0.00	1.53	0.43 ±0.00	0.45 ±0.000	4.65
26	36.72 ±0.06	25.17 ±0.06	3.84	1.09 ±0.01	1.12 ±0.01	2.75	0.35 ±0.00	0.34 ±0.00	2.86	10.11 ±0.06	10 .00±0.06	1.09
27	16.02 ±0.10	13.24 ±0.10	0.53	4.77 ±0.01	4.74 ±0.01	0.63	1.31 ±0.01	1.36 ±0.01	3.82	-	-	-
28	13.17 ±0.10	26.76 ±0.10	2.10	2.26 ±0.01	2.21 ±0.01	2.21	0.73 ±0.03	0.70 ±0.03	4.11	2.89 ±0.03	2.86 ±0.03	1.04

note 1:-: not detect
